# *Mycobacterium paratuberculosis*: A HERV Turn-On for Autoimmunity, Neurodegeneration, and Cancer?

**DOI:** 10.3390/microorganisms12091890

**Published:** 2024-09-13

**Authors:** Coad Thomas Dow, Ellen S. Pierce, Leonardo A. Sechi

**Affiliations:** 1 McPherson Eye Research Institute, University of Wisconsin-Madison, Madison, WI 53705, USA; 2 Independent Researcher, Spokane Valley, WA 99216, USA; ellenpiercemd@gmail.com; 3 Department of Biomedical Science, University of Sassari, 07100 Sassari, Italy; sechila@uniss.it; 4 Azienda Ospedaliera Universitaria di Sassari, Viale San Pietro, 07100 Sassari, Italy

**Keywords:** *Mycobacterium avium* ss. *paratuberculosis* (MAP), human endogenous retrovirus (HERV), type 1 diabetes (T1D), multiple sclerosis (MS), rheumatoid arthritis (RA), amyotrophic lateral sclerosis (ALS), Alzheimer’s disease (AD), Parkinson’s disease (PD), glioblastoma, bromoepiandrosterone (BEA), Temelimab, BNN27

## Abstract

Human endogenous retroviruses (HERVs) are remnants of ancient retroviral infections that, over millions of years, became integrated into the human genome. While normally inactive, environmental stimuli such as infections have contributed to the transcriptional reactivation of HERV-promoting pathological conditions, including the development of autoimmunity, neurodegenerative disease and cancer. What infections trigger HERV activation? *Mycobacterium avium* subspecies *paratuberculosis* (MAP) is a pluripotent driver of human disease. Aside from granulomatous diseases, Crohn’s disease, sarcoidosis and Blau syndrome, MAP is associated with autoimmune disease: type one diabetes (T1D), multiple sclerosis (MS), rheumatoid arthritis (RA) and autoimmune thyroiditis. MAP is also associated with Alzheimer’s disease (AD) and Parkinson’s disease (PD). Autoimmune diabetes, MS and RA are the diseases with the strongest MAP/HERV association. There are several other diseases associated with HERV activation, including diseases whose epidemiology and/or pathology would prompt speculation for a causal role of MAP. These include non-solar uveal melanoma, colon cancer, glioblastoma and amyotrophic lateral sclerosis (ALS). This article further points to MAP infection as a contributor to autoimmunity, neurodegenerative disease and cancer via the un-silencing of HERV. We examine the link between the ever-increasing number of MAP-associated diseases and the MAP/HERV intersection with these diverse medical conditions, and propose treatment opportunities based upon this association.

## 1. Introduction

This article reviews proviral elements that have been incorporated into the human genome and presents the contributory role that these proviral elements play in autoimmunity, neurodegeneration and cancer [1,2,3]. This article reiterates that independent environmental stimuli, particularly infections. may transactivate those endogenous elements and promote these pathogenic conditions. Moreover, it proposes that a particular species of *Mycobacteria* may play a role as an activator of these endogenized viral remnants.

### 1.1. HERV

Human genes encoding proteins make up only 1–2% of the human genome [4]. Elements of exogenous virus infection have been incorporated into animal genomes over millions of years and are represented in the human genome, over 8% of which is comprises these viral remnants [5,6]. First described in 1970, the human endogenous retroviruses (HERVs) were discovered to have originated through ancient retrovirus infection and then incorporated into the germline and passed down through generations via Mendelian inheritance [7,8,9,10]. Repeated independent retroviral infections have generated unique HERV components, leading to more than 100,000 HERV loci in humans; after the initial infections took place, these sequences multiplied themselves using a ‘copy-and-paste’ mechanism known as retrotransposition [11]. While the number of inserted viral elements within the human genome is significant, complete proviral sequences are scarce. Through evolutionary processes involving recombination, deletion, and constant mutational events, many HERV components have been removed from the host genome. As a result, most of these elements are incomplete or carry mutations that render them silenced and incapable of replication [12], Interestingly, a new mechanism has now been found to allow the translation of stop codons from HERV-W at the ribosome level [13].

A notable exception involves two HERV proteins that are expressed fully in human pregnancy; these proteins, syncytins—envelope proteins of HERV-W—have a role in human placental development as well as a specific tolerance to the semi-allogenic fetal tissue to prevent rejection from the mother’s immune system [14,15]. Needless to say, the conservation of “endogenized” retroviral genes that encode these functional proteins is important: the genes that participate in evolutionary biology mark a transition from oviparity to viviparity [16].

HERVs have a genetic structure analogous to that of exogenous retroviruses. They possess two long terminal repeats (LTRs) that enclose the internal coding sequence of the four fundamental retroviral coding domains: *gag*, *pro*, *pol*, and *env* that are exposed to the cellular milieu [17]. HERV nomenclature is somewhat arbitrary: HERV-K represents a group (frequently termed clade) of endogenous retroviruses that utilize lysine transfer RNA for their replication and integration into the genome [18]. Representatives of the HERV-K and HERV-W families are the primary focus of this review. The ancestor of the HERV-W lineage became part of the genome approximately 25 million years ago [19]. This marked the beginning of a crucial evolutionary period [20]. The aforementioned syncytin is the lone fully preserved HERV-W within the human genome [21,22]. The envelope protein of HERV-W (W-ENV) was demonstrated to be immunopathogenic and neurotoxic [12,13,14]. W-ENV was shown to induce pro-inflammatory responses through the Toll-like-receptor 4 cascade [17,18] but also inhibition of oligodendrocyte precursor cell (re)myelination. It can be reactivated by viral infections such a EBV (in MS) and, more recently, SARS-COV-2 [23,24,25].

HERV-K insertions have been more recently added and more than 500 HERV-K elements are dispersed within the human genome [26]. A notable beneficial contribution from the HERV-K family is human endogenous retrovirus K102 (HERV-K102); this is a replication-competent retrovirus associated with foamy macrophages that is unique to humans and has been found to protect against other exogenous viruses as well as some cancers [27]. Moreover, HERV-K102 may play a mechanistic role in other age-related diseases such as atherosclerosis, where the aging steroid metabolome reduces immune protection afforded by HERV-K102 foamy macrophages [28].

The activation, or “un-silencing”, of these genes can be promoted by infection [29]. An example of a microbial trigger of HERV activation is found in autoimmune diabetes (T1D): MAP can activate HERV-W, which plays a role in the etiopathology of T1D [30,31].

### 1.2. Mycobacterium avium Subspecies Paratuberculosis—MAP

*Mycobacterium avium* subspecies *paratuberculosis* (MAP), a unique zoonotic pathogen, poses a significant health risk at the intersection of animals, humans, and the environment [32]. It causes a fatal enteric infectious disease long studied in ruminant animals known as Johne’s disease. Infected animals may not show clinical symptoms for years; yet, during this preclinical stage, the animals shed MAP in their milk and feces. This has led to a “don’t test, don’t tell” scenario in the industry, resulting in an increasing prevalence of Johne’s disease [32]. Moreover, pasteurization does not entirely eliminate MAP from contaminated milk, making it a source of exposure for humans; over 90% of dairy herds in the US have MAP-infected animals [33]. Milk and dairy products are the primary source of MAP infection in humans; pasteurization only partially reduces the MAP load originally present in milk, posing a consumption risk. MAP can also be found in yogurt, cheese, muscle meat, and hamburgers [32]. The same bacterium, MAP, is the putative cause of Crohn’s disease in humans. Countries that were historically free of Johne’s disease acquired it through trade with infected animals, and Crohn’s disease became a lagging indicator of MAP infection [32]. MAP is associated with an expanding list of human diseases, several of which are delineated in this article. Besides Crohn’s disease, the list included sarcoidosis, Blau syndrome, autoimmune diabetes, autoimmune thyroiditis, lupus, multiple sclerosis, rheumatoid arthritis, and Parkinson’s disease [34].

## 2. Autoimmunity

When antigenic tolerance is lost to one’s own self-antigens, the immune response is termed autoimmunity [35]. More than 100 autoimmune diseases have been described and they occur in up to 8% of the population with a higher prevalence in women [36]. Activation of the immune response by HERV can elicit uncontrolled inflammation that subsequently drives chronic inflammation, contributing to the development of autoimmune diseases [37,38]. Mechanisms by which HERV can induce autoimmunity include (1) superantigens, as HERVs can encode proteins that act as superantigens, particularly in CD4 T lymphocytes [39]; (2) molecular mimicry, as autoantigens from ancestral endogenous retroviruses, once un-silenced, can have high homology with eukaryotic elements, triggering an autoimmune response [40,41]; and (3) DNA hypomethylation, as DNA hypomethylation is an epigenetic mechanism of DNA regulation, which is represented by the loss of the methyl group in the 5-methylcytosine nucleotide [42,43] and affects autoimmunity, contributing to the pathogenesis of autoimmunity, such as systemic lupus erythematosus [44,45].

### 2.1. Autoimmune Diabetes (T1D)

Type 1 diabetes (T1D) is an autoimmune disease. The etiology of T1D is incompletely understood but environmental agents are thought to trigger T1D in the genetically at-risk. In the United States, the prevalence of T1D is increasing and is approximately 1 in 300 by 18 years of age. Research into risk factors for T1D is an active area with attempts to identify genetic and environmental triggers that could potentially be targeted for intervention [46].

#### 2.1.1. T1D and MAP

In 2006, Dow postulated that MAP may be an environmental trigger for T1D in the genetically at-risk. Three reasons led to the postulate: (1) shared genetic susceptibilities to both mycobacterial infection and T1D; (2) MAP being the source of the HSP65 protein, providing homology between mycobacterial HSP65 and pancreatic glutamic acid decarboxylase (GAD); and (3) consistent epidemiology findings tying the risk of T1D to early-life exposure to cow’s milk [47,48]. Subsequently, Sechi and associates conducted several studies associating MAP and T1D. They found an association of MAP and T1D patients on their home island of Sardinia [49,50,51]. The island of Sardinia has the second highest incidence of T1D in the world [52]. They found MAP in T1D but not type 2 diabetics [53,54] and in children with T1D [55,56,57]. They confirmed a shared genetic risk linking mycobacterial infection and T1D [58]. They also identified additional MAP peptides that are homologous with pancreatic proteins [51,57,59] and showed that immune reaction to these MAP peptides cross-react with classic islet cell antibodies [60]. Furthermore, they demonstrated parallel findings on the Italian mainland [61,62].

#### 2.1.2. T1D and HERV

Both animal and human studies suggest the involvement of HERV-W in T1D pathogenesis [63]. Sechi drew on his MAP/T1D experience and looked for an additional association with HERV; he found that antibody levels against HERV-W overlapped with or predicted the detection of T1D diagnostic autoantibodies [60]. He and his associates demonstrated that anti-HERV antibodies correlate with seroreactivity against MAP in children at risk of T1D [31].

### 2.2. Multiple Sclerosis

Multiple sclerosis (MS) is an autoimmune demyelinating disease that involves the central nervous system and is characterized by infiltration of myelin-specific CD4+ T cells [64,65]. Identified host genetic risk factors for MS also increase the risk of susceptibility to mycobacterial infections. This includes polymorphisms of the major histocompatibility complex, TLR, vitamin D receptor genes, genes encoding IFN-gamma signaling components and SLC11A1 (also known as natural resistance-associated macrophage protein-1, it is a member of the solute-carrier family) [66,67,68].

#### 2.2.1. MS and MAP

MAP and MS are associated and this too was first studied on the island of Sardinia, where MAP is endemic [69]. The association has also been studied in Japan, where a seroprevalence study confirmed the association between MAP and MS [70]. Supporting the association of MAP and MS is the detection of MAP DNA in the peripheral blood of MS patients [71]. As with T1D, molecular mimicry is felt to play a triggering role in MS. Antibodies against MAP-specific protein MAP_2694295–303 and MAP pentapeptide (MAP_5p) are prevalent in the cerebral spinal fluid (CSF) and serum of those with MS. These peptides have homology with a component of myelin, the myelin basic protein (MBP). MBP is known as a prime target of autoimmune demyelination [72].

#### 2.2.2. MS and HERV

Several investigations provide strong indication of an association between MS and HERV [23,73,74,75,76]. The presence of HERV-W immunoreactivity in active MS lesions is closely linked to areas of active demyelination throughout the progression of lesions in MS brains [77]. Antibodies against specific HERV peptide fragments have been identified in the serum and CSF of patients with MS; HERV-W env-su_93-108_ and HERV-W env-su_248-262_ [78] can distinguish those patients from those with other neurologic diseases.

### 2.3. Rheumatoid Arthritis

Rheumatoid arthritis (RA) is a chronic autoimmune disease featuring pro-inflammatory cytokines that produce erosive joint damage and humoral and cellular immune responses against both self- and infectious peptides [79].

#### 2.3.1. RA and MAP

While the exact cause of RA is unknown, most agree that it is the result of a complex interaction between host genetics and “environmental factors” [80]. The commonly cited infectious agents associated with RA are MAP and the Epstein–Barr virus (EBV) [81]. MAP and EBV are thought to secrete peptides that cross-react against RA-linked self-peptides [79,82,83,84,85].

It was also observed in Iranian patients [86].

#### 2.3.2. RA and HERV

HERV-K viral loads are elevated in RA patients compared to healthy controls; moreover, HERV-K is elevated in the synovial fluid of RA patients compared to patients with osteoarthritis [87]. The pathogenic mechanism of molecular mimicry linking HERV-K to RA has been advanced [88,89]. Specifically, HERV-K env-su_19–37_ has high epitope homology with human antigens [90].

### 2.4. Systemic Lupus Erythematosus

Systemic lupus erythematosus (SLE) is considered a prototypic systemic autoimmune disease and is characterized by autoantibodies and multi-organ involvement [91].

#### 2.4.1. SLE and MAP

The direct association of SLE and MAP is from a single case report [92]. The report shows that immunodominant mycobacterial heat shock protein 65 (HSP65) exuded by MAP shares homology with characteristic anti-Ro and anti-La autoimmune proteins associated with SLE.

#### 2.4.2. SLE and HERV

While the association of MAP and SLE is nascent, the HERV/SLE association is well delineated. Numerous HERV sequences are indeed expressed at altered levels in SLE [93,94]. Both adult and pediatric SLE patients have IgG autoantibodies that recognize the envelope (Env) protein of HERV-K [95]. Immune complexes containing the ERV-K102 envelope protein have the capacity to activate neutrophils, which play a central role in the pathogenesis of SLE. When such complexes are cultured with neutrophils from healthy donors, there is an induction of both neutrophil phagocytosis and neutrophil activation, showing that HERV-K proteins are a target of SLE autoantibodies [96].

## 3. Neurodegeneration

Increasing epidemiologic and experimental data suggest a role for chronic bacterial and viral infections as risk factors for neurodegenerative diseases [97,98,99]. HERVs have repeatedly been implicated in brain disorders [100,101,102], and while this article will primarily discuss the age-related neurodegenerative diseases, it is worth noting the recognition of HERV in the etiopathology of schizophrenia [103,104]. Individuals with schizophrenia can be stratified into groups with differing inflammatory and clinical profiles based upon the presence of HERV-W antigens [105].

Prion-like propagation of misfolded proteins is a hallmark of age-related neurodegenerative diseases [106,107]. HERV un-silencing dramatically increases the dissemination of these protein aggregates, a process that can be inhibited by targeting the HERV protein [108].

### 3.1. Amyotrophic Lateral Sclerosis

Amyotrophic lateral sclerosis (ALS), also known as motor neuron disease (MND), is a progressively degenerative neurological condition that results in the loss of both upper and lower motor neurons located in the primary motor cortex and spinal cord [109,110]. This neuronal loss leads to muscle atrophy, ultimately causing the affected individual to lose control over bodily muscles and essential functions, such as respiration [110,111]. ALS is a highly intricate and diverse disease, characterized by variations in clinical symptoms and disease progression among patients [112]. This complexity makes diagnosing ALS challenging, often requiring patients to wait for up to a year from the onset of symptoms to receive a diagnosis. Currently, the underlying cause of ALS remains unknown, and there is no cure or therapy that can modify the course of the disease; the typical life expectancy for individuals diagnosed with ALS ranges from 2 to 5 years [113,114,115].

#### 3.1.1. ALS and MAP

Reports of ALS clusters in professional rugby players, professional and amateur European soccer players and American football players led Pierce to propose MAP as a dirt-based infectious agent that can be introduced to athletes. The MAP exposure can come via the inhalation of an aerosolized pathogen, or after multiple oral, nasal or subcutaneous introductions of MAP present in the dirt, dust and grass of their playing fields [116]. Pierce et al. followed up with a summary of two published case reports of ALS-like disease that responded to treatment with anti-mycobacterial antibiotics [117].

#### 3.1.2. ALS and HERV

The transactive response DNA-binding protein 43 kDa (TDP-43), which is typically located in the nucleus of neuronal and glial cells, plays a role in RNA regulation. In ALS, TDP-43 is mislocated and aggregated in the cytoplasm, leading to neurodegeneration [118]. The involvement of HERVs in ALS pathology is well recognized and is felt to occur through TDP-43 and neuroinflammatory mediators [119]. Recently, it was reported that Asparaginase-like-1 protein (ASRGL1) (which cleaves isoaspartates, altering protein folding and susceptibility to proteolysis) harbors a copy of the human endogenous retrovirus HML-2, whose overexpression contributes to ALS pathogenesis. In particular, overexpression of HML-2 led to ASRGL1 silencing. Loss of ASRGL1 leading to TDP-43 aggregation is a critical mechanism in ALS pathophysiology [120].

There are HERV-K transcripts found in the post-mortem brains of ALS patients [121]. Moreover, antibodies against a specific HERV peptide, HERV-K env-su19-37, are elevated in both the serum and CSF of patients with ALS and its presence can differentiate ALS from other neurogenerative diseases [78].

### 3.2. Alzheimer’s Disease

Alzheimer’s disease (AD) is the most common cause of dementia, with a worldwide prevalence expecting to rise to greater than 60 million by 2030 and more than 150 million by 2050 [122]. The pathologic hallmarks of AD are extracellular amyloid plaques and intracellular tau tangles, with these pathologies found up to 20 years prior to the onset of dementia [123,124].

#### 3.2.1. AD and MAP

Although no specific agent has been identified as a cause of AD, a number of infectious agents have been linked to AD [99,125,126]. This association is bolstered by the knowledge that AD’s primary pathologic protein, amyloid, is an antimicrobial peptide [127,128]. Mycobacteria in general [129], as well as MAP specifically [130], have been proposed as causal agents of AD. Gaining advantage due to heightened age-related immune risk, i.e., immunosenescence, infection by MAP may subvert cerebral glucose metabolism and exhaust autophagic capacity, both invariant features of AD and mycobacterial infection [129,131,132]. A number of anti-mycobacterial agents that suppress intracellular mycobacterial infection have been found to somewhat favorably impact AD; an epidemiologic study of leprosy patients treated with anti-mycobacterial drugs found the patients to have a significantly lower incidence of AD compared to those untreated [133]. Noteworthy is a proposed treatment of AD with the anti-mycobacterial drug, rifampin; intranasal delivery circumvents the known hepatic toxicity associated with the clearance of systemic rifampin [134,135].

#### 3.2.2. AD and HERV

HERVs have been implicated in the pathogenesis of AD [136,137]. Telescope is a tool that delineates HERV expression and is used to quantify this expression at specific genomic locations [138]. When applied in various datasets, this technology identified HERV-K differential overexpression in AD [139]. An interesting study of blood samples taken before and after individuals transitioned from normal cognition to AD showed what they described as a “storm” of differentially expressed transposable elements [140]. They postulated that this storm induces the immune-mediated neuroinflammation of AD and could be used as a biomarker heralding the phenoconversion from normal cognition to AD [140].

### 3.3. Parkinson’s Disease

Parkinson’s disease (PD) is a common, progressive degenerative disease of the nervous system that manifests clinically by motor symptoms. These symptoms present when the burden of the hallmark aggregated protein inclusion bodies, Lewy bodies, reaches an advanced state [141]. Three lines of research have led to a greatly improved understanding of PD: (1) pathologic staging of the pathology of PD by Braak [142,143], (2) the segregation of initiating PD stimuli to “body first” or “brain first” [144,145], and (3) the identification of polymorphisms of the LRRK2 and PARK genes associated with PD that are also associated with susceptibility to mycobacterial infection and Crohn’s disease [146,147].

#### 3.3.1. PD and MAP

PD is the one neurodegenerative disease in which MAP has been found to play a role, with this link having been initially postulated by Dow [148] and then found by Sechi [149]. The LRRK2 genetic connection to both PD and Crohn’s disease [150,151,152] lends support to this association. Intestinal symptoms are known to precede PD diagnosis [153]. The body-first iteration of PD is, in actuality, gut-first as PD proteinopathy arises in the enteric nervous system and propagates to the brain via the vagus nerve [154,155]. Body-first PD is first seen in the brain within its dorsal motor nucleus of the vagus nerve [156,157]. The historically relevant surgery, truncal vagotomy, is associated with a lower subsequent risk of PD [158]. Brain-first PD is also a misnomer as the PD cerebral pathology comes to the brain via the olfactory nerve [159,160]. Cerebral MAP infection through the olfactory nerve is conceivable; aerosolized MAP may lead to neuro-infection in the same manner of neuro-infection by *Neisseria* that has been demonstrated by olfactory delivery [148,161].

#### 3.3.2. PD and HERV

Wallace et al. used computational biology methods, experimental factor ontology enrichment analysis, to test for associations between HERV-K and various diseases. They found that HERV-K sites were statistically enriched for expressions in PD. This was measured as a false discovery rate (FDR) and they found that the chance of observing a false positive association between HERV-K and PD is 1.8^−9^—that is an FDR of 0.0000000018 [162].

## 4. Cancer

The first description of a cancer releasing HERV viral particles was reported in 1978 and related to testicular cancer [163]. Cell lines from testicular cancer became a model for HERV-K protein expression and its effects on cell function [164]. Since then, HERVs have been associated with a host of cancers [165,166,167]. This includes the hematologic cancers leukemia and multiple myeloma [168]. The diseases featured in the remainder of this section stem from the work of pathologist Ellen Pierce, who coupled facets of MAP epidemiology as well as pathologic features to each of the maladies; in each section, a discussion of MAP epidemiology as it relates to the oncologic malady and HERV is presented.

### 4.1. Uveal Melanoma

Cutaneous melanoma is the most lethal form of skin cancer; if detected and surgically treated early, survival rate is high. After metastasis, survival rates drop significantly [169]. Melanomas arising from the uveal layer in the posterior of the eye are distinct from cutaneous melanomas; while both arise from melanocytes, ultraviolet radiation that triggers cutaneous lesions does have not an equally predominant role in uveal melanomas [170]. Uveal melanomas do not have the molecular changes observed in cutaneous melanomas—changes that are attributable to ultraviolet exposure [171].

#### 4.1.1. Uveal Melanoma and MAP

While there is no direct evidence of MAP and uveal melanoma, Pierce et al. note the high association of uveal melanoma with farming-related agricultural occupations [172]. They identify a MAP-contaminated farm environment as a source of MAP exposure [172] where MAP persists in the farm soil, dust and water [173]. Farmers have an increased risk of developing uveal melanoma [174,175]. Pierce et al. describes a report of three clusters or “geospatial accumulations” of uveal melanoma and ascribes environmental MAP exposure to these clusters. They further posit MAP infection and resultant inflammation as a mechanism of cancer induction [176]. They describe the microenvironment of uveal melanoma as an inflammatory phenotype that prevents an efficient antitumor response [177]. They relate the estimated 80–90% exogenous environment factors associated with cancer and note the “hit and run” manner in which prior infections can trigger cancer [178].

#### 4.1.2. Uveal Melanoma and HERV

A role of HERV in melanoma has been identified [179]. Mechanistically, HERV-D expression mediates intracellular fusion of melanoma cells that may drive the genetic changes that promote tumor growth [180]. The HERV association with melanoma appears to extend to both cutaneous and ocular melanoma as it is reported that a peptide from HERV is detected in both cutaneous and uveal melanoma tissues but not in normal tissues [181].

### 4.2. Colon Cancer

In 2020, colorectal cancer was the third most common cancer and second leading cause of cancer deaths worldwide [182]. The inflammatory bowel diseases Crohn’s and ulcerative colitis are associated with colon cancer [183,184]. Crohn’s disease has long been associated with MAP [185] and there is also a MAP association, but less so, with ulcerative colitis [186].

#### 4.2.1. Colon Cancer and MAP

MAP organisms have been identified in the intestines of patients with sporadic colorectal cancer where high-magnification oil-immersion light microscopy was used (×1000 total magnification rather than the usual 400× total magnification) [187]. MAP can cause acute and chronic intestinal goblet cell hyperplasia, an early pathologic feature of sporadic colon cancer, leading Pierce to suggest that the persistent presence of MAP may result in not only inflammatory bowel diseases but also colon cancer [188].

#### 4.2.2. Colon Cancer and HERV

There are several unique HERV-H loci expressed in colorectal cancer samples both from pathologic tissues and from cell lines [189,190]. The presence of the products of these specific HERV-related genes found in peripheral blood samples suggests a utility of their use as tumor biomarkers for these patients [191].

### 4.3. Glioblastoma

Glioblastoma is the most common primary brain tumor of adults; even with aggressive treatment, median survival is only 15 months [192]. Infectious agents have been proposed as causes of nervous system cancers; this includes viruses [193,194] and the parasite *Toxoplasma gondii* [195]; there have been two case reports relating *Toxoplasma gondii* to glioblastoma [196,197]. There is a notably increased rate of gliomas in rural residents who live in areas with domestic livestock [198,199,200,201]. While pesticide use was suspected, it was dismissed in favor of a proposed zoonotic microorganism as the responsible agent for the increased rate in farm residents and workers [202,203]. Interestingly, there is an increased rate of glioblastoma in tennis players, baseball pitchers and infielders, as well as umpires [204]. Pierce suggests a pathologic equivalence between the pseudopalisading necrosis of gliomas and similar pathologic features of hypoxic mycobacterial granulomas [204,205].

#### 4.3.1. Glioblastoma and MAP

Pierce proposes MAP as a mycobacterial infectious agent that is diffusely distributed in the soil [173] and, as such, provides a source for routine exposure to individuals in those environments [204]. Moreover, Pierce proposes that MAP is a promotor of the defining histopathologic changes in glioblastoma: endothelial cell proliferation and pseudopalisading necrosis [204].

#### 4.3.2. Glioblastoma and HERV

An array of differentially expressed HERV elements are found in glioblastoma [206]. HML-2, a subtype of the HERV-K family, is expressed in a number of human cancers, including glioblastoma [192,207]. HERV-K overexpression is associated with glioma stem cells and an aggressive phenotype; also, down-regulation of this expression with CRISPR results in decreased HERV transcription [208].

## 5. Treating MAP/HERV

Recognizing a MAP/HERV association may open therapeutic pathways to treatment. Discussed here only in passing are anti-mycobacterial antibiotic treatments that are being used in Crohn’s disease [209]. Rather, what is presented is an anti-viral treatment for HERV-associated disease, as well as the potential of “upstream” treatment of the steroid metabolome and how that may favorably affect both MAP infection and HERV-associated disease.

An antiviral against HERV, GNbAC1 (now called Temelimab), is interestingly registered in clinical trials for both multiple sclerosis (NCT02782858, NCT01639300, NCT03239860, NCT04480307) [210] and autoimmune diabetes (NCT03179423); these are the two diseases with the greatest MAP/HERV association (as discussed in Section 2). Temelimab is an IgG4 monoclonal antibody that has been developed to specifically target HERV-W-Env and to neutralize the effect of HERV-W-Env; this therapeutic effort extends to HERV-W related cancers [211].

Both drugs and vaccines may find increasing utility in the treatment of these diseases [166]. Anti-viral drugs are proposed in the neurodegenerative proteinopathies [108]. Anti-HIV retroviral drugs have demonstrated some benefit in ALS and they decrease HERV-K load after 24 weeks of therapy [212]. A strategy to augment tumor antigenicity via HERV-induced inflammation is another approach to fight cancer [167], including gliomas [213]. Moreover, it has been suggested that various tumors will be responsive to vaccines against HERV-K Gag [214]. Thus, HERV-targeting agents could provide a broad new class of therapeutics for autoimmunity, neurodegeneration and cancer.

An “upstream” approach to treat both mycobacterial infection and HERV-driven disease is proposed with analogs of dehydroepiandrosterone, DHEA. The levels of DHEA decrease and the levels of cortisol increase with age and this ratio is associated with immunosenescence [215,216]. Direct DHEA supplementation is mostly ineffective [217,218].

BNN27 is a new DHEA derivative without unwanted steroidogenic effects; this agent has shown promise in animal studies [219]. HE2000, a synthetic analog of DHEA, found utility in the pre-antiviral drug era of HIV/AIDS [220]. It is a non-androgenic, non-anabolic steroid also known as BEA (16-bromoepiandrosterone). By acting to restore homeostasis to dysregulated metabolic/immune signaling networks, BEA may provide a more youthful metabolome and immune phenotype [221], as demonstrated with an improved response to tuberculosis infection [222,223,224,225].

Cortisol may trigger HERV-associated diseases [27,28]; thus, efforts to develop a DHEA analog to increase the DHEA/cortisol ratio reminiscent of youth may alleviate many of the diseases presented in this article by restoring immune and metabolic homeostasis. Nonetheless, testing the levels of autoantibodies against the envelope of different HERVs that may be reactivated in MAP-associated pathologies is of primary importance since these have been shown to be linked to a higher level of protection and a better outcome of the disease [226].

## 6. Conclusions

By any account, MAP has been a formidable adversary for animal agriculture and animal and human health. Known for more than a century as the cause of Johne’s disease, this fatal infection has continued to expand around the globe [32,33,227]. MAP has insidiously spread among animals and to humans by way of food, water, dirt and air. MAP can participate in the granuloma of granulomatous diseases and trigger autoimmune diseases by providing homologous epitopes to human self-proteins. This article proposes another mean by which MAP causes disease: inflammation caused by MAP infection and persistency may initiate the activation of long dormant viral elements—HERVs—carried in our DNA previously silenced by DNA modifications (methylation, etc.), which, when reawakened, transcribe pathogenic proteins such as the envelope of the retroviruses which contribute to inflammation and autoimmune response in different human diseases.

## References

[1] Grandi N., Tramontano E. (2018). Human endogenous retroviruses are ancient acquired elements still shaping innate immune responses. Front. Immunol..

[2] Dopkins N., Nixon D.F. (2023). Activation of human endogenous retroviruses and its physiological consequences. Nat. Rev. Mol. Cell Biol..

[3] Bannert N., Hofmann H., Block A., Hohn O. (2018). HERVs new role in cancer: From accused perpetrators to cheerful protectors. Front. Microbiol..

[4] Lander E.S., Linton L.M., Birren B., Nusbaum C., Zody M.C., Baldwin J., Devon K., Dewar K., Doyle M., FitzHugh W. (2001). Initial sequencing and analysis of the human genome. Nature.

[5] De Parseval N., Heidmann T. (2005). Human endogenous retroviruses: From infectious elements to human genes. Cytogenet. Genome Res..

[6] Hughes J.F., Coffin J.M. (2005). Human endogenous retroviral elements as indicators of ectopic recombination events in the primate genome. Genetics.

[7] Venter J.C., Adams M.D., Myers E.W., Li P.W., Mural R.J., Sutton G.G., Smith H.O., Yandell M., Evans C.A., Holt R.A. (2001). The sequence of the human genome. Science.

[8] Weiss R.A. (2006). The discovery of endogenous retroviruses. Retrovirology.

[9] Griffiths D.J. (2001). Endogenous retroviruses in the human genome sequence. Genome Biol..

[10] Patience C., Wilkinson D.A., Weiss R.A. (1997). Our retroviral heritage. Trends Genet..

[11] Thomas J., Perron H., Feschotte C. (2018). Variation in proviral content among human genomes mediated by LTR recombination. Mobile DNA.

[12] Mager D.L., Goodchild N.L. (1989). Homologous recombination between the LTRs of a human retrovirus-like element causes a 5-kb deletion in two siblings. Am. J. Hum. Genet..

[13] Brunel J., Paganini J., Galloux M., CHARVET B., Perron H. (2024). An HERV-W ENV transcription in atypical memory B cells linked to COVID-19 evolution and risk for long COVID can express the encoded protein from a ribosome readthrough of mRNA from chromosome X. medRxiv.

[14] Noorali S., Rotar I.C., Lewis C., Pestaner J.P., Pace D.G., Sison A., Bagasra O. (2009). Role of HERV-W syncytin-1 in placentation and maintenance of human pregnancy. Appl. Immunohistochem. Mol. Morphol..

[15] Xiang Y., Liang H. (2021). The regulation and functions of endogenous retrovirus in embryo development and stem cell differentiation. Stem Cells Int..

[16] Dupressoir A., Lavialle C., Heidmann T. (2012). From ancestral infectious retroviruses to bona fide cellular genes: Role of the captured syncytins in placentation. Placenta.

[17] Vargiu L., Rodriguez-Tomé P., Sperber G.O., Cadeddu M., Grandi N., Blikstad V., Tramontano E., Blomberg J. (2016). Classification and characterization of human endogenous retroviruses; mosaic forms are common. Retrovirology.

[18] Gifford R.J., Blomberg J., Coffin J.M., Fan H., Heidmann T., Mayer J., Stoye J., Tristem M., Johnson W.E. (2018). Nomenclature for endogenous retrovirus (ERV) loci. Retrovirology.

[19] Costas J. (2002). Characterization of the intragenomic spread of the human endogenous retrovirus family HERV-W. Mol. Biol. Evol..

[20] Voisset C., Blancher A., Perron H., Mandrand B., Mallet F., Paranhos-Baccalà G. (1999). Phylogeny of a novel family of human endogenous retrovirus sequences, HERV-W, in humans and other primates. AIDS Res. Hum. Retroviruses.

[21] Cheynet V., Ruggieri A., Oriol G., Blond J.L., Boson B., Vachot L., Verrier B., Cosset F.L., Mallet F. (2005). Synthesis, assembly, and processing of the Env ERVWE1/syncytin human endogenous retroviral envelope. J. Virol..

[22] Voisset C., Bouton O., Bedin F., Duret L., Mandrand B., Mallet F., Paranhos-Baccala G. (2000). Chromosomal distribution and coding capacity of the human endogenous retrovirus HERV-W family. AIDS Res. Hum. Retroviruses.

[23] Cossu D., Tomizawa Y., Sechi L.A., Hattori N. (2023). Epstein–Barr Virus and Human Endogenous Retrovirus in Japanese Patients with Autoimmune Demyelinating Disorders. Int. J. Mol. Sci..

[24] Arru G., Caggiu E., Leoni S., Mameli G., Pugliatti M., Sechi G.P., Sechi L.A. (2015). Natalizumab modulates the humoral response against HERV-Wenv73-88 in a follow-up study of Multiple Sclerosis patients. J. Neurol. Sci..

[25] Pérez-Pérez S., Domínguez-Mozo M.I., García-Martínez M., Ballester-González R., Nieto-Gañán I., Arroyo R., Alvarez-Lafuente R. (2022). Epstein-Barr Virus Load Correlates with Multiple Sclerosis-Associated Retrovirus Envelope Expression. Biomedicines.

[26] Schön U., Diem O., Leitner L., Günzburg W.H., Mager D.L., Salmons B., Leib-Mösch C. (2009). Human endogenous retroviral long terminal repeat sequences as cell type-specific promoters in retroviral vectors. J. Virol..

[27] Laderoute M.P. (2015). A new paradigm about HERV-K102 particle production and blocked release to explain cortisol mediated immunosenescence and age-associated risk of chronic disease. Discov. Med..

[28] Laderoute M. (2020). The paradigm of immunosenescence in atherosclerosis-cardiovascular disease (ASCVD). Discov. Med..

[29] Castro F.L.d., Brustolini O.J.B., Geddes V.E.V., Souza J.P.B.M.d., Alves-Leon S.V., Aguiar R.S., Vasconcelos A.T.R. (2022). Modulation of HERV Expression by Four Different Encephalitic Arboviruses during Infection of Human Primary Astrocytes. Viruses.

[30] Noli M., Meloni G., Manca P., Cossu D., Palermo M., Sechi L.A. (2021). HERV-W and Mycobacterium avium subspecies paratuberculosis Are at Play in Pediatric Patients at Onset of Type 1 Diabetes. Pathogens.

[31] Niegowska M., Wajda-Cuszlag M., Stępień-Ptak G., Trojanek J., Michałkiewicz J., Szalecki M., Sechi L.A. (2019). Anti-HERV-WEnv antibodies are correlated with seroreactivity against Mycobacterium avium subsp. paratuberculosis in children and youths at T1D risk. Sci. Rep..

[32] Dow C.T., Alvarez B.L. (2022). Mycobacterium paratuberculosis zoonosis is a One Health emergency. EcoHealth.

[33] Lombard J.E., Gardner I.A., Jafarzadeh S.R., Fossler C.P., Harris B., Capsel R.T., Wagner B.A., Johnson W.O. (2013). Herd-level prevalence of Mycobacterium avium subsp. paratuberculosis infection in United States dairy herds in 2007. Prev. Vet. Med..

[34] Ekundayo T.C., Okoh A.I. (2020). Systematic assessment of Mycobacterium avium subspecies paratuberculosis infections from 1911-2019: A growth analysis of association with human autoimmune diseases. Microorganisms.

[35] Wang L., Wang F.S., Gershwin M.E. (2015). Human autoimmune diseases: A comprehensive update. J. Intern. Med..

[36] Angum F., Khan T., Kaler J., Siddiqui L., Hussain A. (2020). The prevalence of autoimmune disorders in women: A narrative review. Cureus.

[37] Hurst T.P., Magiorkinis G. (2015). Activation of the innate immune response by endogenous retroviruses. J. Gen. Virol..

[38] Stearrett N., Dawson T., Rahnavard A. (2021). Expression of human endogenous retroviruses in systemic lupus erythematosus: Multiomic integration with gene expression. Front. Immunol..

[39] Posnett D.N., Yarilina A.A. (2001). Sleeping with the enemy—Endogenous superantigens in humans. Immunity.

[40] Parthasarathy R., Wakefield D., Santiago F.S., Kaakoush N.O., Tedla N. (2024). Horizontal gene transfer and endogenous retroviruses as mechanisms for molecular mimicry. Lancet Microbe.

[41] Gröger V., Cynis H. (2018). Human endogenous retroviruses and their putative role in the development of autoimmune disorders such as multiple sclerosis. Front. Microbiol..

[42] Wilson A.S., Power B.E., Molloy P.L. (2007). DNA hypomethylation and human diseases. Biochim. Biophys. Acta (BBA)-Rev. Cancer.

[43] Canas C.A., Canas F., Bonilla-Abadia F., Ospina F.E., Tobón G.J. (2016). Epigenetics changes associated to environmental triggers in autoimmunity. Autoimmunity.

[44] Renaudineau Y., Youinou P. (2011). Epigenetics and autoimmunity, with special emphasis on methylation. Keio J. Med..

[45] Hughes T., Sawalha A.H. (2011). The role of epigenetic variation in the pathogenesis of systemic lupus erythematosus. Arthritis Res. Ther..

[46] Maahs D.M., West N.A., Lawrence J.M., Mayer-Davis E.J. (2010). Epidemiology of type 1 diabetes. Endocrinol. Metab. Clin..

[47] Dow C.T. (2006). Paratuberculosis and Type I diabetes: Is this the trigger?. Med. Hypotheses.

[48] Dow C.T. (2018). Failure of TRIGR Study Opens Door to Alternative Explanation of T1DM Etiopathology. J. Diabetes Metab..

[49] Sechi L.A., Rosu V., Pacifico A., Fadda G., Ahmed N., Zanetti S. (2008). Humoral immune responses of type 1 diabetes patients to Mycobacterium avium subsp. paratuberculosis lend support to the infectious trigger hypothesis. Clin. Vaccine Immunol. CVI.

[50] Sechi L.A., Paccagnini D., Salza S., Pacifico A., Ahmed N., Zanetti S. (2008). Mycobacterium avium subspecies paratuberculosis bacteremia in type 1 diabetes mellitus: An infectious trigger?. Clin. Infect. Dis. Off. Publ. Infect. Dis. Soc. Am..

[51] Cossu A., Rosu V., Paccagnini D., Cossu D., Pacifico A., Sechi L.A. (2011). MAP3738c and MptD are specific tags of Mycobacterium avium subsp. paratuberculosis infection in type I diabetes mellitus. Clin. Immunol..

[52] Songini M., Mannu C., Targhetta C., Bruno G. (2017). Type 1 diabetes in Sardinia: Facts and hypotheses in the context of worldwide epidemiological data. Acta Diabetol..

[53] Rosu V., Ahmed N., Paccagnini D., Pacifico A., Zanetti S., Sechi L.A. (2008). Mycobacterium avium subspecies paratuberculosis is not associated with type-2 diabetes mellitus. Ann. Clin. Microbiol. Antimicrob..

[54] Rosu V., Ahmed N., Paccagnini D., Gerlach G., Fadda G., Hasnain S.E., Zanetti S., Sechi L.A. (2009). Specific immunoassays confirm association of Mycobacterium avium Subsp. paratuberculosis with type-1 but not type-2 diabetes mellitus. PLoS ONE.

[55] Manca Bitti M.L., Masala S., Capasso F., Rapini N., Piccinini S., Angelini F., Pierantozzi A., Lidano R., Pietrosanti S., Paccagnini D. (2012). Mycobacterium avium subsp. paratuberculosis in an Italian cohort of type 1 diabetes pediatric patients. J. Immunol. Res..

[56] Cossu A., Ferrannini E., Fallahi P., Antonelli A., Sechi L.A. (2013). Antibodies recognizing specific Mycobacterium avium subsp. paratuberculosis’s MAP3738c protein in type 1 diabetes mellitus children are associated with serum Th1 (CXCL10) chemokine. Cytokine.

[57] Masala S., Zedda M.A., Cossu D., Ripoli C., Palermo M., Sechi L.A. (2013). Zinc transporter 8 and MAP3865c homologous epitopes are recognized at T1D onset in Sardinian children. PLoS ONE.

[58] Paccagnini D., Sieswerda L., Rosu V., Masala S., Pacifico A., Gazouli M., Ikonomopoulos J., Ahmed N., Zanetti S., Sechi L.A. (2009). Linking chronic infection and autoimmune diseases: Mycobacterium avium subspecies paratuberculosis, SLC11A1 polymorphisms and type-1 diabetes mellitus. PLoS ONE.

[59] Masala S., Paccagnini D., Cossu D., Brezar V., Pacifico A., Ahmed N., Mallone R., Sechi L.A. (2011). Antibodies recognizing Mycobacterium avium paratuberculosis epitopes cross-react with the beta-cell antigen ZnT8 in Sardinian type 1 diabetic patients. PLoS ONE.

[60] Niegowska M., Paccagnini D., Mannu C., Targhetta C., Songini M., Sechi L.A. (2016). Recognition of ZnT8, proinsulin, and homologous MAP peptides in Sardinian children at risk of T1D precedes detection of classical islet antibodies. J. Diabetes Res..

[61] Masala S., Cossu D., Piccinini S., Rapini N., Massimi A., Porzio O., Pietrosanti S., Lidano R., Bitti M.L.M., Sechi L.A. (2014). Recognition of zinc transporter 8 and MAP3865c homologous epitopes by new-onset type 1 diabetes children from continental Italy. Acta Diabetol..

[62] Masala S., Cossu D., Piccinini S., Rapini N., Mameli G., Manca Bitti M.L., Sechi L.A. (2015). Proinsulin and MAP3865c homologous epitopes are a target of antibody response in new-onset type 1 diabetes children from continental Italy. Pediatr. Diabetes.

[63] Levet S., Medina J., Joanou J., Demolder A., Queruel N., Réant K., Normand M., Seffals M., Dimier J., Germi R. (2017). An ancestral retroviral protein identified as a therapeutic target in type-1 diabetes. JCI Insight.

[64] Olsson T., Barcellos L.F., Alfredsson L. (2017). Interactions between genetic, lifestyle and environmental risk factors for multiple sclerosis. Nat. Rev. Neurol..

[65] Ota K., Matsui M., Milford E.L., Mackin G.A., Weiner H.L., Hafler D.A. (1990). T-cell recognition of an immuno-dominant myelin basic protein epitope in multiple sclerosis. Nature.

[66] Goris A., Vandebergh M., McCauley J.L., Saarela J., Cotsapas C. (2022). Genetics of multiple sclerosis: Lessons from polygenicity. Lancet Neurol..

[67] Wasko N.J., Nichols F., Clark R.B. (2020). Multiple sclerosis, the microbiome, TLR2, and the hygiene hypothesis. Autoimmun. Rev..

[68] Cossu D., Masala S., Cocco E., Paccagnini D., Tranquilli S., Frau J., Marrosu M.G., Sechi L.A. (2013). Association of Mycobacterium avium subsp. paratuberculosis and SLC11A1 polymorphisms in Sardinian multiple sclerosis patients. J. Infect. Dev. Ctries..

[69] Cossu D., Yokoyama K., Hattori N. (2018). Bacteria–host interactions in multiple sclerosis. Front. Microbiol..

[70] Cossu D., Cocco E., Paccagnini D., Masala S., Ahmed N., Frau J., Marrosu M.G., Sechi L.A. (2011). Association of Mycobacterium avium subsp. paratuberculosis with multiple sclerosis in Sardinian patients. PLoS ONE.

[71] Cossu D., Yokoyama K., Sechi L.A., Otsubo S., Tomizawa Y., Momotani E., Hattori N. (2016). Humoral response against host-mimetic homologous epitopes of Mycobacterium avium subsp. paratuberculosis in Japanese multiple sclerosis patients. Sci. Rep..

[72] Cossu D., Yokoyama K., Hattori N. (2017). Conflicting role of Mycobacterium species in multiple sclerosis. Front. Neurol..

[73] Christensen T. (2017). Human endogenous retroviruses in the aetiology of MS. Acta Neurol. Scand..

[74] Mafi S., Savadi Oskoee D., Bannazadeh Baghi H., Azadi A., Ahangar Oskouee M. (2023). Association of Epstein–Barr Virus (EBV) and Human Endogenous Retroviruses (HERV) with Multiple Sclerosis in Northwest of Iran. Int. J. Inflamm..

[75] Ruberto S., Cossu D., Sechi L.A. (2023). Correlation between antibodies against the pathogenic pHERV-W envelope protein and the inflammatory phase of multiple sclerosis. Immunology.

[76] Laska M.J., Brudek T., Nissen K.K., Christensen T., Møller-Larsen A., Petersen T., Nexø B.A. (2012). Expression of HERV-Fc1, a human endogenous retrovirus, is increased in patients with active multiple sclerosis. J. Virol..

[77] van Horssen J., van der Pol S., Nijland P., Amor S., Perron H. (2016). Human endogenous retrovirus W in brain lesions: Rationale for targeted therapy in multiple sclerosis. Mult. Scler. Relat. Disord..

[78] Arru G., Mameli G., Deiana G., Rassu A., Piredda R., Sechi E., Caggiu E., Bo M., Nako E., Urso D. (2018). Humoral immunity response to human endogenous retroviruses K/W differentiates between amyotrophic lateral sclerosis and other neurological diseases. Eur. J. Neurol..

[79] Bo M., Erre G.L., Niegowska M., Piras M., Taras L., Longu M.G., Passiu G., Sechi L.A. (2018). Interferon regulatory factor 5 is a potential target of autoimmune response triggered by Epstein-barr virus and Mycobacterium avium subsp. paratuberculosis in rheumatoid arthritis: Investigating a mechanism of molecular mimicry. Clin. Exp. Rheumatol..

[80] McInnes I.B., Schett G. (2011). The pathogenesis of rheumatoid arthritis. N. Engl. J. Med..

[81] Bo M., Jasemi S., Uras G., Erre G.L., Passiu G., Sechi L.A. (2020). Role of Infections in the Pathogenesis of Rheumatoid Arthritis: Focus on Mycobacteria. Microorganisms.

[82] Bo M., Arru G., Niegowska M., Erre G.L., Manchia P.A., Sechi L.A. (2019). Association between Lipoprotein Levels and Humoral Reactivity to Mycobacterium avium subsp. paratuberculosis in Multiple Sclerosis, Type 1 Diabetes Mellitus and Rheumatoid Arthritis. Microorganisms.

[83] Bo M., Erre G.L., Bach H., Slavin Y.N., Manchia P.A., Passiu G., Sechi L.A. (2019). PtpA and PknG Proteins Secreted by Mycobacterium avium subsp. paratuberculosis are Recognized by Sera from Patients with Rheumatoid Arthritis: A Case-Control Study. J. Inflamm. Res..

[84] Jasemi S., Erre G.L., Cadoni M.L., Bo M., Sechi L.A. (2021). Humoral Response to Microbial Biomarkers in Rheumatoid Arthritis Patients. J. Clin. Med..

[85] Erre G.L., Cossu D., Masala S., Mameli G., Cadoni M.L., Serdino S., Longu M.G., Passiu G., Sechi L.A. (2014). Mycobacterium tuberculosis lipoarabinomannan antibodies are associated to rheumatoid arthritis in Sardinian patients. Clin. Rheumatol..

[86] Asgari N., Ghaemi E.A., Tavasoli S., Aghaei M., Razavi Nikoo H., Sechi L.A., Zamani S. (2023). Detection of Mycobacterium avium Subspecies paratuberculosis in the Blood of Patients with Rheumatoid Arthritis by Using Serological and Molecular Techniques. Curr. Microbiol..

[87] Reynier F., Verjat T., Turrel F., Imbert P.E., Marotte H., Mougin B., Miossec P. (2009). Increase in human endogenous retrovirus HERV-K (HML-2) viral load in active rheumatoid arthritis. Scand. J. Immunol..

[88] Freimanis G., Hooley P., Ejtehadi H.D., Ali H.A., Veitch A., Rylance P.B., Alawi A., Axford J., Nevill A., Murray P.G. (2010). A role for human endogenous retrovirus-K (HML-2) in rheumatoid arthritis: Investigating mechanisms of pathogenesis. Clin. Exp. Immunol..

[89] Nelson P.N., Roden D., Nevill A., Freimanis G.L., Trela M., Ejtehadi H.D., Bowman S., Axford J., Veitch A.M., Tugnet N. (2014). Rheumatoid arthritis is associated with IgG antibodies to human endogenous retrovirus gag matrix: A potential pathogenic mechanism of disease?. J. Rheumatol..

[90] Mameli G., Erre G.L., Caggiu E., Mura S., Cossu D., Bo M., Cadoni M.L., Piras A., Mundula N., Colombo E. (2017). Identification of a HERV-K env surface peptide highly recognized in Rheumatoid Arthritis (RA) patients: A cross-sectional case-control study. Clin. Exp. Immunol..

[91] Rose J. (2022). Autoimmune connective tissue diseases: Systemic lupus erythematosus and rheumatoid arthritis. Emerg. Med. Clinics.

[92] Dow C.T. (2016). Detection of *M. paratuberculosis* Bacteremia in a Child with Lupus Erythematosus and Sjogren’s Syndrome. Autoimmune Infect. Dis..

[93] Tokuyama M., Kong Y., Song E., Jayewickreme T., Kang I., Iwasaki A. (2018). ERVmap analysis reveals genome-wide transcription of human endogenous retroviruses. Proc. Natl. Acad. Sci. USA.

[94] Iñiguez L.P., de Mulder Rougvie M., Stearrett N., Jones R.B., Ormsby C.E., Reyes-Terán G., Crandall K.A., Nixon D.F., Bendall M.L. (2019). Transcriptomic analysis of human endogenous retroviruses in systemic lupus erythematosus. Proc. Natl. Acad. Sci. USA.

[95] Khadjinova A.I., Wang X., Laine A., Ukadike K., Eckert M., Stevens A., Bengtsson A.A., Lood C., Mustelin T. (2022). Autoantibodies against the envelope proteins of endogenous retroviruses K102 and K108 in patients with systemic lupus erythematosus correlate with active disease. Clin. Exp. Rheumatol..

[96] Tokuyama M., Gunn B.M., Venkataraman A., Kong Y., Kang I., Rakib T., Townsend M.J., Costenbader K.H., Alter G., Iwasaki A. (2021). Antibodies against human endogenous retrovirus K102 envelope activate neutrophils in systemic lupus erythematosus. J. Exp. Med..

[97] De Chiara G., Marcocci M.E., Sgarbanti R., Civitelli L., Ripoli C., Piacentini R., Garaci E., Grassi C., Palamara A.T. (2012). Infectious agents and neurodegeneration. Mol. Neurobiol..

[98] Lotz S.K., Blackhurst B.M., Reagin K.L., Funk K.E. (2021). Microbial infections are a risk factor for neurodegenerative diseases. Front. Cell. Neurosci..

[99] Norins L.C. (2021). Licensed anti-microbial drugs logical for clinical trials against pathogens currently suspected in Alzheimer’s disease. Antibiotics.

[100] Römer C. (2021). Viruses and Endogenous Retroviruses as Roots for Neuroinflammation and Neurodegenerative Diseases. Front. Neurosci..

[101] Gruchot J., Lewen I., Dietrich M., Reiche L., Sindi M., Hecker C., Herrero F., Charvet B., Weber-Stadlbauer U., Hartung H.P. (2023). Transgenic expression of the HERV-W envelope protein leads to polarized glial cell populations and a neurodegenerative environment. Proc. Natl. Acad. Sci. USA.

[102] Dolei A., Uleri E., Ibba G., Caocci M., Piu C., Serra C. (2015). The aliens inside human DNA: HERV-W/MSRV/syncytin-1 endogenous retroviruses and neurodegeneration. J. Infect. Dev. Ctries..

[103] Gruchot J., Herrero F., Weber-Stadlbauer U., Meyer U., Küry P. (2023). Interplay between activation of endogenous retroviruses and inflammation as common pathogenic mechanism in neurological and psychiatric disorders. Brain. Behav. Immun..

[104] Karlsson H., Bachmann S., Schröder J., McArthur J., Torrey E.F., Yolken R.H. (2001). Retroviral RNA identified in the cerebrospinal fluids and brains of individuals with schizophrenia. Proc. Natl. Acad. Sci. USA.

[105] Tamouza R., Meyer U., Foiselle M., Richard J.R., Wu C.L., Boukouaci W., Le Corvoisier P., Barrau C., Lucas A., Perron H. (2021). Identification of inflammatory subgroups of schizophrenia and bipolar disorder patients with HERV-W ENV antigenemia by unsupervised cluster analysis. Transl. Psychiatry.

[106] Scialò C., De Cecco E., Manganotti P., Legname G. (2019). Prion and Prion-Like Protein Strains: Deciphering the Molecular Basis of Heterogeneity in Neurodegeneration. Viruses.

[107] O’Carroll A., Coyle J., Gambin Y. (2020). Prions and Prion-like assemblies in neurodegeneration and immunity: The emergence of universal mechanisms across health and disease. Semin. Cell Dev. Biol..

[108] Liu S., Heumüller S.E., Hossinger A., Müller S.A., Buravlova O., Lichtenthaler S.F., Denner P., Vorberg I.M. (2023). Reactivated endogenous retroviruses promote protein aggregate spreading. Nat. Commun..

[109] Chiò A., Traynor B.J. (2015). Motor neuron disease in 2014. Biomarkers for ALS--in search of the Promised Land. Nat. Rev. Neurol..

[110] Foster L.A., Salajegheh M.K. (2019). Motor Neuron Disease: Pathophysiology, Diagnosis, and Management. Am. J. Med..

[111] Kiernan M.C. (2018). Motor neuron disease in 2017: Progress towards therapy in motor neuron disease. Nat. Rev. Neurol..

[112] Bendotti C., Bonetto V., Pupillo E., Logroscino G., Al-Chalabi A., Lunetta C., Riva N., Mora G., Lauria G., Weishaupt J.H. (2020). Focus on the heterogeneity of amyotrophic lateral sclerosis. Amyotroph. Lateral Scler. Front. Degener..

[113] Hardiman O., van den Berg L.H., Kiernan M.C. (2011). Clinical diagnosis and management of amyotrophic lateral sclerosis. Nat. Rev. Neurol..

[114] Chiò A., Logroscino G., Hardiman O., Swingler R., Mitchell D., Beghi E., Traynor B.G. (2009). Prognostic factors in ALS: A critical review. Amyotroph. Lateral. Scler..

[115] Longinetti E., Fang F. (2019). Epidemiology of amyotrophic lateral sclerosis: An update of recent literature. Curr. Opin. Neurol..

[116] Pierce E.S. (2018). How did Lou Gehrig get Lou Gehrig’s disease? Mycobacterium avium subspecies paratuberculosis in manure, soil, dirt, dust and grass and amyotrophic lateral sclerosis (motor neurone disease) clusters in football, rugby and soccer players. Med. Hypotheses.

[117] Pierce E.S., Barkhaus P., Beauchamp M., Bromberg M., Carter G.T., Goslinga J., Greeley D., Kihuwa-Mani S., Levitsky G., Lund I. (2022). ALSUntangled# 66: Antimycobacterial antibiotics. Amyotroph. Lateral Scler. Front. Degener..

[118] Prasad A., Bharathi V., Sivalingam V., Girdhar A., Patel B.K. (2019). Molecular mechanisms of TDP-43 misfolding and pathology in amyotrophic lateral sclerosis. Front. Mol. Neurosci..

[119] Dubowsky M., Theunissen F., Carr J.M., Rogers M.-L. (2023). The Molecular Link Between TDP-43, Endogenous Retroviruses and Inflammatory Neurodegeneration in Amyotrophic Lateral Sclerosis: A Potential Target for Triumeq, an Antiretroviral Therapy. Mol. Neurobiol..

[120] Garcia-Montojo M., Fathi S., Rastegar C., Simula E.R., Doucet-O’Hare T., Cheng Y.H.H., Abrams R.P.M., Pasternack N., Malik N., Bachani M. (2024). TDP-43 proteinopathy in ALS is triggered by loss of ASRGL1 and associated with HML-2 expression. Nat. Commun..

[121] Douville R., Liu J., Rothstein J., Nath A. (2011). Identification of active loci of a human endogenous retrovirus in neurons of patients with amyotrophic lateral sclerosis. Ann. Neurol..

[122] Li X., Feng X., Sun X., Hou N., Han F., Liu Y. (2022). Global, regional, and national burden of Alzheimer’s disease and other dementias, 1990–2019. Front. Aging Neurosci..

[123] Sutphen C.L., Jasielec M.S., Shah A.R., Macy E.M., Xiong C., Vlassenko A.G., Benzinger T.L., Stoops E.E., Vanderstichele H.M., Brix B. (2015). Longitudinal cerebrospinal fluid biomarker changes in preclinical Alzheimer disease during middle age. JAMA Neurol..

[124] Rafii M.S., Aisen P.S. (2023). Detection and treatment of Alzheimer’s disease in its preclinical stage. Nature Aging.

[125] Bulgart H.R., Neczypor E.W., Wold L.E., Mackos A.R. (2020). Microbial involvement in Alzheimer disease development and progression. Mol. Neurodegener..

[126] Lophatananon A., Carr M., Mcmillan B., Dobson C., Itzhaki R., Parisi R., Ashcroft D.M., Muir K.R. (2023). The association of herpes zoster and influenza vaccinations with the risk of developing dementia: A population-based cohort study within the UK Clinical Practice Research Datalink. BMC Public. Health.

[127] Soscia S.J., Kirby J.E., Washicosky K.J., Tucker S.M., Ingelsson M., Hyman B., Burton M.A., Goldstein L.E., Duong S., Tanzi R.E. (2010). The Alzheimer’s disease-associated amyloid β-protein is an antimicrobial peptide. PLoS ONE.

[128] Blanchard R.H. (1917). Liability and Compensation Insurance; Industrial Accidents and Their Prevention, Employers’ Liability, Workmen’s Compensation, Insurance of Employers’ Liability and Workmen’s Compensation.

[129] Broxmeyer L. (2005). Thinking the unthinkable: Alzheimer’s, Creutzfeldt–Jakob and Mad Cow disease: The age-related reemergence of virulent, Foodborne, bovine tuberculosis or losing your mind for the sake of a shake or burger. Med. Hypotheses.

[130] Dow C.T. (2021). Warm, sweetened milk at the twilight of immunity—Alzheimer’s disease—Inflammaging, insulin resistance, *M. paratuberculosis* and immunosenescence. Front. Immunol..

[131] De la Monte S.M., Wands J.R. (2008). Alzheimer’s disease is type 3 diabetes—Evidence reviewed. J. Diabetes Sci. Technol..

[132] Cai Z., Zhao B., Li K., Zhang L., Li C., Quazi S.H., Tan Y. (2012). Mammalian target of rapamycin: A valid therapeutic target through the autophagy pathway for Alzheimer’s disease?. J. Neurosci. Res..

[133] McGeer P.L., Harada N., Kimura H., McGeer E.G., Schulzer M. (1992). Prevalence of dementia amongst elderly Japanese with leprosy: Apparent effect of chronic drug therapy. Dement. Geriatr. Cogn. Disord..

[134] Umeda T., Tanaka A., Sakai A., Yamamoto A., Sakane T., Tomiyama T. (2018). Intranasal rifampicin for Alzheimer’s disease prevention. Alzheimer’s Dement. Transl. Res. Clin. Interv..

[135] Dow C.T., Kidess L. (2023). Proposing Intranasal Rifampin for Alzheimer’s Disease and the Other Age-Related Neurodegenerative Proteinopathies. Preprints.

[136] Sun W., Samimi H., Gamez M., Zare H., Frost B. (2018). Pathogenic tau-induced piRNA depletion promotes neuronal death through transposable element dysregulation in neurodegenerative tauopathies. Nat. Neurosci..

[137] Grundman J., Spencer B., Sarsoza F., Rissman R.A. (2021). Transcriptome analyses reveal tau isoform-driven changes in transposable element and gene expression. PLoS ONE.

[138] Bendall M.L., De Mulder M., Iñiguez L.P., Lecanda-Sánchez A., Pérez-Losada M., Ostrowski M.A., Jones R.B., Mulder L.C., Reyes-Terán G., Crandall K.A. (2019). Telescope: Characterization of the retrotranscriptome by accurate estimation of transposable element expression. PLoS Comput. Biol..

[139] Dawson T., Rentia U., Sanford J., Cruchaga C., Kauwe J.S., Crandall K.A. (2023). Locus specific endogenous retroviral expression associated with Alzheimer’s disease. Front. Aging Neurosci..

[140] Macciardi F., Giulia Bacalini M., Miramontes R., Boattini A., Taccioli C., Modenini G., Malhas R., Anderlucci L., Gusev Y., Gross T.J. (2022). A retrotransposon storm marks clinical phenoconversion to late-onset Alzheimer’s disease. GeroScience.

[141] Gaig C., Tolosa E. (2009). When does Parkinson’s disease begin?. Mov. Disord..

[142] Braak H., Del Tredici K., Rüb U., De Vos R.A., Steur E.N.J., Braak E. (2003). Staging of brain pathology related to sporadic Parkinson’s disease. Neurobiol. Aging.

[143] Chen H., Wang K., Scheperjans F., Killinger B. (2022). Environmental triggers of Parkinson’s disease–Implications of the Braak and dual-hit hypotheses. Neurobiol. Dis..

[144] Horsager J., Knudsen K., Sommerauer M. (2022). Clinical and imaging evidence of brain-first and body-first Parkinson’s disease. Neurobiol. Dis..

[145] Horsager J., Andersen K.B., Knudsen K., Skjærbæk C., Fedorova T.D., Okkels N., Schaeffer E., Bonkat S.K., Geday J., Otto M. (2020). Brain-first versus body-first Parkinson’s disease: A multimodal imaging case-control study. Brain.

[146] Schurr E., Alcais A., Singh M., Mehra N., Abel L. (2007). Mycobacterial infections: PARK2 and PACRG associations in leprosy. Tissue Antigens.

[147] Hur E.-M., Lee B.D. (2021). LRRK2 at the Crossroad of Aging and Parkinson’s Disease. Genes.

[148] Dow C.T. (2014). *M. paratuberculosis* and Parkinson’s disease--is this a trigger. Med. Hypotheses.

[149] Arru G., Caggiu E., Paulus K., Sechi G.P., Mameli G., Sechi L.A. (2016). Is there a role for Mycobacterium avium subspecies paratuberculosis in Parkinson’s disease?. J. Neuroimmunol..

[150] Herrick M.K., Tansey M.G. (2021). Is LRRK2 the missing link between inflammatory bowel disease and Parkinson’s disease?. Npj Park. Dis..

[151] Derkinderen P., Neunlist M. (2018). Crohn’s and Parkinson disease: Is LRRK2 lurking around the corner?. Nat. Rev. Gastroenterol. Hepatol..

[152] Hui K.Y., Fernandez-Hernandez H., Hu J., Schaffner A., Pankratz N., Hsu N.-Y., Chuang L.-S., Carmi S., Villaverde N., Li X. (2018). Functional variants in the LRRK2 gene confer shared effects on risk for Crohn’s disease and Parkinson’s disease. Sci. Transl. Med..

[153] Konings B., Villatoro L., Van den Eynde J., Barahona G., Burns R., McKnight M., Hui K., Yenokyan G., Tack J., Pasricha P.J. (2023). Gastrointestinal syndromes preceding a diagnosis of Parkinson’s disease: Testing Braak’s hypothesis using a nationwide database for comparison with Alzheimer’s disease and cerebrovascular diseases. Gut.

[154] Ulusoy A., Rusconi R., Pérez-Revuelta B.I., Musgrove R.E., Helwig M., Winzen-Reichert B., Monte D.A.D. (2013). Caudo-rostral brain spreading of α-synuclein through vagal connections. EMBO Mol. Med..

[155] Zhang W., Ye Y., Song J., Sang T., Xia T., Xie L., Qiu X., Zeng Q., Luo X. (2023). Research Progress of Microbiota-Gut-Brain Axis in Parkinson’s Disease. J. Integr. Neurosci..

[156] Borghammer P., Van Den Berge N. (2019). Brain-first versus gut-first Parkinson’s disease: A hypothesis. J. Parkinsons Dis..

[157] Chandra R., Sokratian A., Chavez K.R., King S., Swain S.M., Snyder J.C., West A.B., Liddle R.A. (2023). Gut mucosal cells transfer α-synuclein to the vagus nerve. JCI Insight.

[158] Svensson E., Horváth-Puhó E., Thomsen R.W., Djurhuus J.C., Pedersen L., Borghammer P., Sørensen H.T. (2015). Vagotomy and subsequent risk of P arkinson’s disease. Ann. Neurol..

[159] Wiseman J.A., Murray H.C., Faull R.L., Dragunow M., Turner C.P., Dieriks B.V., Curtis M.A. (2024). Aggregate-prone brain regions in Parkinson’s disease are rich in unique N-terminus α-synuclein conformers with high proteolysis susceptibility. Npj Park. Dis..

[160] Stefani A., Iranzo A., Holzknecht E., Perra D., Bongianni M., Gaig C., Heim B., Serradell M., Sacchetto L., Garrido A. (2021). Alpha-synuclein seeds in olfactory mucosa of patients with isolated REM sleep behaviour disorder. Brain.

[161] Sjölinder H., Jonsson A.-B. (2010). Olfactory nerve—A novel invasion route of Neisseria meningitidis to reach the meninges. PLoS ONE.

[162] Wallace A.D., Wendt G.A., Barcellos L.F., De Smith A.J., Walsh K.M., Metayer C., Costello J.F., Wiemels J.L., Francis S.S. (2018). To ERV is human: A phenotype-wide scan linking polymorphic human endogenous retrovirus-K insertions to complex phenotypes. Front. Genet..

[163] Bronson D.L., Ritzi D.M., Fraley E.E., Dalton A.J. (1978). Morphologic evidence for retrovirus production by epithelial cells derived from a human testicular tumor metastasis. J. Natl. Cancer Inst..

[164] Stricker E., Peckham-Gregory E.C., Scheurer M.E. (2023). HERVs and Cancer-A Comprehensive Review of the Relationship of Human Endogenous Retroviruses and Human Cancers. Biomedicines.

[165] Kassiotis G. (2014). Endogenous retroviruses and the development of cancer. J. Immunol..

[166] Hosseiniporgham S., Sechi L.A. (2023). Anti-HERV-K drugs and vaccines, possible therapies against tumors. Vaccines.

[167] Jansz N., Faulkner G.J. (2021). Endogenous retroviruses in the origins and treatment of cancer. Genome Biol..

[168] Li T., Qian K., Han J., Liu Y., Jia L., Wang X., Li T., Zhang B., Li J., Li H. (2023). Higher Expression of Human Endogenous Retrovirus-K was Observed in Peripheral B Lymphocytes of Leukemia and Lymphoma Patients. AIDS Res. Hum. Retroviruses.

[169] Davis L.E., Shalin S.C., Tackett A.J. (2019). Current state of melanoma diagnosis and treatment. Cancer Biol. Ther..

[170] de Lange M.J., Razzaq L., Versluis M., Verlinde S., Dogrusoz M., Bohringer S., Marinkovic M., Luyten G.P., de Keizer R.J., de Gruijl F.R. (2015). Distribution of GNAQ and GNA11 Mutation Signatures in Uveal Melanoma Points to a Light Dependent Mutation Mechanism. PLoS ONE.

[171] Amaro A., Gangemi R., Piaggio F., Angelini G., Barisione G., Ferrini S., Pfeffer U. (2017). The biology of uveal melanoma. Cancer Metastasis Rev..

[172] Pierce E.S., Jindal C., Choi Y.M., Efird J.T. (2023). The evidence for Mycobacterium avium subspecies paratuberculosis (MAP) as a cause of nonsolar uveal melanoma: A narrative review. Transl. Cancer Res..

[173] Rhodes G., Henrys P., Thomson B.C., Pickup R.W. (2013). Mycobacterium avium subspecies paratuberculosis is widely distributed in British soils and waters: Implications for animal and human health. Environ. Microbiol..

[174] Gallagher R.P. (1988). Ocular melanoma in farmers. Am. J. Ind. Med..

[175] Pearce N., Reif J.S. (1990). Epidemiologic studies of cancer in agricultural workers. Am. J. Ind. Med..

[176] Parsonnet J. (1995). Bacterial infection as a cause of cancer. Environ. Health Perspect..

[177] Bronkhorst I.H., Jager M.J. (2013). Inflammation in uveal melanoma. Eye.

[178] Lax A.J., Thomas W. (2002). How bacteria could cause cancer: One step at a time. Trends Microbiol..

[179] Buscher K., Trefzer U., Hofmann M., Sterry W., Kurth R., Denner J. (2005). Expression of human endogenous retrovirus K in melanomas and melanoma cell lines. Cancer Res..

[180] Huang G., Li Z., Wan X., Wang Y., Dong J. (2013). Human endogenous retroviral K element encodes fusogenic activity in melanoma cells. J. Carcinog..

[181] Schiavetti F., Thonnard J., Colau D., Boon T., Coulie P.G. (2002). A human endogenous retroviral sequence encoding an antigen recognized on melanoma by cytolytic T lymphocytes. Cancer Res..

[182] Sung H., Ferlay J., Siegel R.L., Laversanne M., Soerjomataram I., Jemal A., Bray F. (2021). Global Cancer Statistics 2020: GLOBOCAN Estimates of Incidence and Mortality Worldwide for 36 Cancers in 185 Countries. CA Cancer J. Clin..

[183] Kim E.R., Chang D.K. (2014). Colorectal cancer in inflammatory bowel disease: The risk, pathogenesis, prevention and diagnosis. World J. Gastroenterol..

[184] Choi P.M., Zelig M.P. (1994). Similarity of colorectal cancer in Crohn’s disease and ulcerative colitis: Implications for carcinogenesis and prevention. Gut.

[185] Kuenstner J.T., Naser S., Chamberlin W., Borody T., Graham D.Y., McNees A., Hermon-Taylor J., Hermon-Taylor A., Dow C.T., Thayer W. (2017). The Consensus from the Mycobacterium avium ssp. paratuberculosis (MAP) Conference 2017. Front Public Health.

[186] Pierce E.S. (2010). Ulcerative colitis and Crohn’s disease: Is Mycobacterium avium subspecies paratuberculosis the common villain?. Gut Pathog..

[187] Jeyanathan M., Alexander D.C., Turenne C.Y., Girard C., Behr M.A. (2006). Evaluation of in situ methods used to detect Mycobacterium avium subsp. paratuberculosis in samples from patients with Crohn’s disease. J. Clin. Microbiol..

[188] Pierce E.S. (2018). Could Mycobacterium avium subspecies paratuberculosis cause Crohn’s disease, ulcerative colitis...and colorectal cancer?. Infect. Agent. Cancer.

[189] Alves P.M., Lévy N., Stevenson B.J., Bouzourene H., Theiler G., Bricard G., Viatte S., Ayyoub M., Vuilleumier H., Givel J.-C.R. (2008). Identification of tumor-associated antigens by large-scale analysis of genes expressed in human colorectal cancer. Cancer Immun..

[190] Pérot P., Mullins C.S., Naville M., Bressan C., Hühns M., Gock M., Kühn F., Volff J.-N., Trillet-Lenoir V., Linnebacher M. (2015). Expression of young HERV-H loci in the course of colorectal carcinoma and correlation with molecular subtypes. Oncotarget.

[191] Tavakolian S., Iranshahi M., Faghihloo E. (2023). The Evaluation of HERV-K np9, rec, gag Expression in Isolated Human Peripheral Blood Mononuclear Cell (PBMC) of Gastric and Colon Cancer. Adv. Biomed. Res..

[192] Bondy M.L., Scheurer M.E., Malmer B., Barnholtz-Sloan J.S., Davis F.G., Il’yasova D., Kruchko C., McCarthy B.J., Rajaraman P., Schwartzbaum J.A. (2008). Brain tumor epidemiology: Consensus from the Brain Tumor Epidemiology Consortium. Cancer.

[193] Holdhoff M., Guner G., Rodriguez F.J., Hicks J.L., Zheng Q., Forman M.S., Ye X., Grossman S.A., Meeker A.K., Heaphy C.M. (2017). Absence of Cytomegalovirus in Glioblastoma and Other High-grade Gliomas by Real-time PCR, Immunohistochemistry, and In Situ Hybridization. Clin. Cancer Res. Off. J. Am. Assoc. Cancer Res..

[194] Akhtar S., Vranic S., Cyprian F.S., Al Moustafa A.E. (2018). Epstein-Barr Virus in Gliomas: Cause, Association, or Artifact?. Front. Oncol..

[195] Thirugnanam S., Rout N., Gnanasekar M. (2013). Possible role of Toxoplasma gondii in brain cancer through modulation of host microRNAs. Infect. Agent. Cancer.

[196] Schuman L.M., Choi N.W., Gullen W.H. (1967). Relationship of central nervous system neoplasms to Toxoplasma gondii infection. Am. J. Public Health Nation’s Health.

[197] MacDonald A.B. (2022). Borrelia Infected Neoplastic Glial Cells in Five Patients with Glioblastoma Multiformae. Med. Clin. Sci..

[198] Reif J., Pearce N., Fraser J. (1989). Cancer risks in New Zealand farmers. Int. J. Epidemiol..

[199] Reif J.S., Pearce N., Fraser J. (1989). Occupational risks for brain cancer: A New Zealand Cancer Registry-based study. J. Occup. Med. Off. Publ. Ind. Med. Assoc..

[200] Morrison H.I., Semenciw R.M., Morison D., Magwood S., Mao Y. (1992). Brain cancer and farming in western Canada. Neuroepidemiology.

[201] Choi N.W., Schuman L.M., Gullen W.H. (1970). Epidemiology of primary central nervous system neoplasms. I. Mortality from primary central nervous system neoplasms in Minnesota. Am. J. Epidemiol..

[202] Carozza S.E., Li B., Elgethun K., Whitworth R. (2008). Risk of childhood cancers associated with residence in agriculturally intense areas in the United States. Environ. Health Perspect..

[203] Efird J.T., Davies S.W., O’Neal W.T., Anderson E.J. (2014). Animal viruses, bacteria, and cancer: A brief commentary. Front. Public. Health.

[204] Pierce E.S. (2019). Baseballs, tennis balls, livestock farm manure, the IDH1 mutation, endothelial cell proliferation and hypoxic pseudopalisading (granulomatous) necrosis: Mycobacterium avium subspecies paratuberculosis and the epidemiology, cellular metabolism and histology of diffuse gliomas, including glioblastoma. Open Vet. J..

[205] Shah K.K., Pritt B.S., Alexander M.P. (2017). Histopathologic review of granulomatous inflammation. J. Clin. Tuberc. Other Mycobact. Dis..

[206] Yuan Z., Yang Y., Zhang N., Soto C., Jiang X., An Z., Zheng W.J. (2021). Human Endogenous Retroviruses in Glioblastoma Multiforme. Microorganisms.

[207] Xue B., Sechi L.A., Kelvin D.J. (2020). Human Endogenous Retrovirus K (HML-2) in Health and Disease. Front. Microbiol..

[208] Shah A.H., Rivas S.R., Doucet-O’Hare T.T., Govindarajan V., DeMarino C., Wang T., Ampie L., Zhang Y., Banasavadi-Siddegowda Y.K., Walbridge S. (2023). Human endogenous retrovirus K contributes to a stem cell niche in glioblastoma. J. Clin. Investig..

[209] Agrawal G., Clancy A., Huynh R., Borody T. (2020). Profound remission in Crohn’s disease requiring no further treatment for 3-23 years: A case series. Gut Pathog..

[210] Hartung H.P., Derfuss T., Cree B.A., Sormani M.P., Selmaj K., Stutters J., Prados F., MacManus D., Schneble H.M., Lambert E. (2022). Efficacy and safety of temelimab in multiple sclerosis: Results of a randomized phase 2b and extension study. Mult. Scler..

[211] Stricker E., Peckham-Gregory E.C., Scheurer M.E. (2023). CancerHERVdb: Human Endogenous Retrovirus (HERV) Expression Database for Human Cancer Accelerates Studies of the Retrovirome and Predictions for HERV-Based Therapies. J. Virol..

[212] Mochan A. (2019). HIV related motor neuron disease/syndrome: The—Potentially treatable—Retroviral link in ALS?. J. Neurol. Sci..

[213] Ramsoomair C.K., Ceccarelli M., Heiss J.D., Shah A.H. (2023). The epitranscriptome of high-grade gliomas: A promising therapeutic target with implications from the tumor microenvironment to endogenous retroviruses. J. Transl. Med..

[214] Kraus B., Fischer K., Sliva K., Schnierle B.S. (2014). Vaccination directed against the human endogenous retrovirus-K (HERV-K) gag protein slows HERV-K gag expressing cell growth in a murine model system. Virol. J..

[215] Buford T.W., Willoughby D.S. (2008). Impact of DHEA(S) and cortisol on immune function in aging: A brief review. Appl. Physiol. Nutr. Metab..

[216] Baylis D., Bartlett D.B., Syddall H.E., Ntani G., Gale C.R., Cooper C., Lord J.M., Sayer A.A. (2013). Immune-endocrine biomarkers as predictors of frailty and mortality: A 10-year longitudinal study in community-dwelling older people. Age.

[217] Schwartz A.G. (2022). Dehydroepiandrosterone, Cancer, and Aging. Aging Dis..

[218] Klinge C.M., Clark B.J., Prough R.A. (2018). Dehydroepiandrosterone Research: Past, Current, and Future. Vitam. Horm..

[219] Fragkiadaki E., Katsanou L., Vartzoka F., Gravanis A., Pitsikas N. (2024). Effects of low doses of the novel dehydroepiandrosterone (DHEA) derivative BNN27 in rat models of anxiety. Psychopharmacology.

[220] Reading C., Dowding C., Schramm B., Garsd A., Onizuka-Handa N., Stickney D., Frincke J. (2006). Improvement in immune parameters and human immunodeficiency virus-1 viral response in individuals treated with 16alpha-bromoepiandrosterone (HE2000). Clin. Microbiol. Infect..

[221] Seddon J.A., Chiang S.S., Esmail H., Coussens A.K. (2018). The Wonder Years: What Can Primary School Children Teach Us About Immunity to Mycobacterium tuberculosis?. Front. Immunol..

[222] Stickney D.R., Noveljic Z., Garsd A., Destiche D.A., Frincke J.M. (2007). Safety and activity of the immune modulator HE2000 on the incidence of tuberculosis and other opportunistic infections in AIDS patients. Antimicrob. Agents Chemother..

[223] López-Torres M.O., Marquina-Castillo B., Ramos-Espinosa O., Mata-Espinosa D., Barrios-Payan J.A., Baay-Guzman G., Yepez S.H., Bini E., Torre-Villalvazo I., Torres N. (2021). 16α-Bromoepiandrosterone as a new candidate for experimental diabetes-tuberculosis co-morbidity treatment. Clin. Exp. Immunol..

[224] Bongiovanni B., Mata-Espinosa D., D’Attilio L., Leon-Contreras J.C., Marquez-Velasco R., Bottasso O., Hernandez-Pando R., Bay M.L. (2015). Effect of cortisol and/or DHEA on THP1-derived macrophages infected with Mycobacterium tuberculosis. Tuberculosis.

[225] Bongiovanni B., Díaz A., Santucci N., D’Attilio L.D., Bottasso O., Hernández Pando R., Bay M.L. (2022). The Immunoregulatory Actions of DHEA in Tuberculosis, A Tool for Therapeutic Intervention?. Front. Endocrinol..

[226] Garcia-Montojo M., Simula E.R., Fathi S., McMahan C., Ghosal A., Berry J.D., Cudkowicz M., Elkahloun A., Johnson K., Norato G. (2022). Antibody Response to HML-2 May Be Protective in Amyotrophic Lateral Sclerosis. Ann. Neurol..

[227] Duarte L., Santos-Reis M., Cunha M.V. (2023). Widespread circulation and transmission risk of Mycobacterium avium subsp. paratuberculosis at the livestock-wildlife-environment interface in a Mediterranean agro-forestry farmstead. Environ. Pollut..

